# Structural MRI in Frontotemporal Dementia: Comparisons between Hippocampal Volumetry, Tensor-Based Morphometry and Voxel-Based Morphometry

**DOI:** 10.1371/journal.pone.0052531

**Published:** 2012-12-20

**Authors:** Miguel Ángel Muñoz-Ruiz, Päivi Hartikainen, Juha Koikkalainen, Robin Wolz, Valtteri Julkunen, Eini Niskanen, Sanna-Kaisa Herukka, Miia Kivipelto, Ritva Vanninen, Daniel Rueckert, Yawu Liu, Jyrki Lötjönen, Hilkka Soininen

**Affiliations:** 1 Institute of Clinical Medicine, Neurology, University of Eastern Finland, Kuopio, Finland; 2 Department of Neurology, Kuopio University Hospital, Kuopio, Finland; 3 Knowledge Intensive Services, VTT Technical Research Centre of Finland, Tampere, Finland; 4 Biomedical Image Analysis Group, Department of Computing, Imperial College London, London, United Kingdom; 5 Department of Clinical Radiology, Kuopio University Hospital, Kuopio, Finland; 6 Aging Research Centre, Karolinska Institutet, Stockholm, Sweden; Banner Alzheimer’s Institute, United States of America

## Abstract

**Background:**

MRI is an important clinical tool for diagnosing dementia-like diseases such as Frontemporal Dementia (FTD). However there is a need to develop more accurate and standardized MRI analysis methods.

**Objective:**

To compare FTD with Alzheimer’s Disease (AD) and Mild Cognitive Impairment (MCI) with three automatic MRI analysis methods - Hippocampal Volumetry (HV), Tensor-based Morphometry (TBM) and Voxel-based Morphometry (VBM), in specific regions of interest in order to determine the highest classification accuracy.

**Methods:**

Thirty-seven patients with FTD, 46 patients with AD, 26 control subjects, 16 patients with progressive MCI (PMCI) and 48 patients with stable MCI (SMCI) were examined with HV, TBM for shape change, and VBM for gray matter density. We calculated the Correct Classification Rate (CCR), sensitivity (SS) and specificity (SP) between the study groups.

**Results:**

We found unequivocal results differentiating controls from FTD with HV (hippocampus left side) (CCR = 0.83; SS = 0.84; SP = 0.80), with TBM (hippocampus and amygdala (CCR = 0.80/SS = 0.71/SP = 0.94), and with VBM (all the regions studied, especially in lateral ventricle frontal horn, central part and occipital horn) (CCR = 0.87/SS = 0.81/SP = 0.96). VBM achieved the highest accuracy in differentiating AD and FTD (CCR = 0.72/SS = 0.67/SP = 0.76), particularly in lateral ventricle (frontal horn, central part and occipital horn) (CCR = 0.73), whereas TBM in superior frontal gyrus also achieved a high accuracy (CCR = 0.71/SS = 0.68/SP = 0.73). TBM resulted in low accuracy (CCR = 0.62) in the differentiation of AD from FTD using all regions of interest, with similar results for HV (CCR = 0.55).

**Conclusion:**

Hippocampal atrophy is present not only in AD but also in FTD. Of the methods used, VBM achieved the highest accuracy in its ability to differentiate between FTD and AD.

## Introduction

After Alzheimer’s disease (AD), frontotemporal lobar degeneration is the most frequent cause of dementia in middle-aged individuals [1]. The highest prevalence has been observed in two studies performed in the UK in persons between 45 and 64 years, with prevalence rates between 15 and 15.5 per 100,000 inhabitants [1], [2]. It also occurs, though less frequently, among older people as reported in a 66–75-year old cohort in a northern Italian study (78 per 100.000) [3], and in a cohort based on 85 year olds subjects in a Swedish study (prevalence of the frontal variant of 3%) [4].

Brain imaging methods provide useful information on the diagnosis of frontotemporal lobar degeneration [5]. There are patterns of neuroimaging findings that can differentiate frontotemporal dementia (FTD) from AD and from other frontotemporal lobar degeneration syndromes [6].

Frontotemporal lobar degeneration is a general term that includes several disorders that share certain common characteristics, e.g. the main areas affected are the frontal and temporal lobes and these diseases display similar clinical symptoms, of which FTD is the most frequent. FTD can be divided into three clinical syndromes; the behavioral variant in frontotemporal dementia and two language variants: progressive nonfluent aphasia and semantic dementia [5]. These clinical syndromes also correlate with specific patterns of brain atrophy [7], [8].

FTD patients are usually younger than other dementia patients [9]. The brains of FTD patients show a tendency towards more extensive atrophy than in the normal population, but they are less affected than the brains of AD patients [10], [11]. A direct comparison identified a similar pattern of regional volumetric differences in AD patients compared with age-matched FTD patients, although of lower magnitude [12].

Behavioral variant FTD, the most common of the three syndromes, is associated with variable frontal and temporal lobe atrophy and non-affected posterior cortical areas [13]. The atrophy may be asymmetrical [9], [14]–[17], often with a right-side predominance which helps to differentiate this condition from semantic dementia, where the temporal lobe affection is predominantly on the left side [13], [18]. In some other cases, patients with behavioral variant FTD have displayed remarkable atrophy in the anterior temporal lobe with a less significant presence in the frontal regions [17]. Some studies have indicated that behavioral variant FTD involves also other structures such as thalamus [7], [9], [16], [19], striatum [9], [16], [19], and hypothalamus [20]. One study [7] also emphasized the importance of the reduction in white matter in the superior longitudinal fasciculus, inferior longitudinal fasciculus and in the inferior fronto-occipital fasciculus, attributable to damaged interconnections between the different brain cortices.

Whole-brain atrophy comparisons between FTD and AD have revealed no remarkable results, but analysis of regional brain atrophy has been more successful [8]. The frontal regions affected in FTD are spared in AD until the late stages of the disease, whereas hippocampus and entorhinal cortex exhibit atrophy early in disease progression [21]. Some studies have reported correlations with autopsal findings, where brain atrophy in behavioral variant FTD patients was detected in the anterior cingulate, frontal insula, subcallosal gyrus, and striatum, bilaterally [22] and hypothalamus [20], whereas AD patients displayed more extensive atrophy in the posterior parietal and occipital cortices [22], and in posterior cingulate and medial temporal lobe [23]. Differentiating AD patients from controls and subjects with mild cognitive impairment (MCI) who will convert to AD from those who will remain stable can be undertaken with a high degree of sensitivity and specificity by hippocampal volumetry (HV) [24], [25] since this technique assesses hippocampus atrophy. However, hippocampal atrophy is not restricted to AD [26], but can also be detected in FTD.

These findings obtained from structural MRI are increasingly becoming critical steps in the clinical differential diagnosis of dementia diseases but still today, no consensus has been reached over which is the most efficient analysis method. Several new automatic approaches, in addition to visual evaluation and the “gold standard” manual volumetry have been postulated. A huge amount of data has been acquired, such as in the recent investigations involving large cohorts of controls, MCI and AD subjects, the Alzheimer’s Disease Neuroimaging Study (ADNI) (http://www.adni-info.org/) and ADDNeuroMed [24]. As part of the PredictAD project, we have analysed MRI data from the ADNI cohort. We studied the accuracy of different analysis methods such as cortical thickness measurement, hippocampal volumetry, hippocampus atrophy, manifold-based learning and tensor-based morphometry (TBM) to differentiate AD from stable mild cognitive impairment (SMCI), progressive MCI (PMCI) and controls [27]–[29].

Voxel-based morphometry (VBM) provides objectivity in brain measurement and takes into account the whole brain [30], [31]. The procedure consists of comparing gray matter, white matter and cerebrospinal fluid density between groups of subjects on a voxel-by-voxel basis. It has been used in patients with dementia, other settings [32], [33] and healthy ageing subjects [34]. A recent VBM study indicated that behavioral variant FTD could be divided into four anatomically different subtypes, two associated with temporal lobe volume loss and the other two subtypes linked with frontal lobe volume loss [17]. Earlier studies have reported atrophy in the orbitofrontal cortex, dorsolateral prefrontal cortex, left premotor cortex, anterior insula, anterior cingulate cortex [9], [15], [16], left inferior temporal gyrus [9], amygdala [16] and the hippocampus [9], [16].

In TBM analyses, morphometric differences are measured by registering the MRI scans of subjects to a common reference space, and then analyzing the resulting non-linear deformation fields [35], [36]. This technique has proven useful for monitoring the progression of atrophy in various neurodegenerative diseases [8], [23], [37], [38].

Here we extend this work to include patients with FTD; in the present paper we will focus on HV, TBM, and VBM in order to identify the highest accuracy for differentiating FTD patients from controls and from patients with AD and MCI. The classification study was performed using regions of interest (ROI)-wise analysis. HV is a well validated biomarker for AD and its combination with other volumes can improve accuracy [29]. Regional volumes are more powerful than regional thicknesses in classifying AD from controls and MCI stages. We hypothesized that compared to the standard biomarker HV, the automatic whole brain measurements would increase accuracy. We hoped that these methods could provide data that could help to achieve a better clinical differential diagnosis in the dementia diseases.

## Materials and Methods

### Subjects

A total of 173 subjects were included in this study. The demographic and clinical data are shown in *Table 1*.

**Table 1:** Demographic and clinical data of study groups.

	C	FTD	AD	SMCI	PMCI	P-values
**Number of subjects**	26	37	46	48	16	
**Gender male/female**	12/14	20/17	14/32	18/30	7/9	.257^E^
**Age in years**	74±4(66–81)	66±9(41–80)	74±6(58–88)	73±5(63–82)	72±6(55–78)	.000^F^
**Education in years**	7±2(4–12)	8±4^A^(4–20)	8±3(4–20)	NA	NA	.268^G^
**MMSE**	27±2(24–30)	20±7^B^(6–29)	20±4(9–24)	26±2^C^(23–30)	25±3^D^(20–30)	.000^H^
**Total Hippocampal Volumes (ml)**	4346±499(3535–5865)	3788±620(2588–5712)	3440±741(2028–5662)	4191±527(3288–5262)	3741±437(2976–4717)	.000^ I^
**Normalized Hippocampal Volumes** **(zero mean, unit standard deviation)**	0.69±0.65(−0.50–2.40)	−0.48±0.98(−2.85–1.76)	−0.52±1.11(−2.71–2.23)	0.56±0.59(−0.73–1.69)	−0.21±0.61(−1.20–0.95)	.000^J^

C = Healthy controls, FTD = Frontotemporal dementia, AD = Alzheimer’s disease, SMCI = Stable mild cognitive impairment and PMCI = Progressive mild cognitive impairment, MMSE = Mini Mental State Examination.

Results are expressed as mean ± standard deviation, range in parentheses.

A 33 subjects in Education in years,

B 34 subjects in MMSE,

C 45 subjects in MMSE,

D 15 subjects in MMSE,

E Pearson Chi-Square test,

F One-way Anova,

G Kruskal-Wallis test,

H One-way Anova. The statistical significance of differences at the.05 level.

I, J Results of One-way Anova of the study groups. Results are expressed as mean ± standard deviation, with 95% confidence interval in parentheses.

#### FTD patients

There were 37 patients who fulfilled the clinical diagnostic criteria of FTD [5] and for whom we had technically valid brain images after the quality control of images. All FTD patients were clinically examined in the Kuopio University Hospital by an experienced neurologist (P.H.). Twenty-seven of the FTD patients were originally recruited to the rare dementias project, and 10 cases to the Demspect project. The clinical diagnosis of all FTD patients was ascertained after at least three years’ clinical follow-up that consisted of a thorough clinical history, physical examination, neuropsychological testing, blood tests, as well as brain MRI and single photon emission tomography at entry. In four cases, FTD patients displayed the clinical syndrome of behavioural variant FTD combined with signs of motoneuron disease. In three cases, FTD patients had a mutation in the C9ORF72 gene. Nine cases underwent autopsy to obtain a pathological confirmation of frontotemporal lobar degeneration with TDP (TAR DNA-binding protein of 43 kDa) positive brain immunohistochemistry. MRI images and neuropsychological tests of the FTD patients were performed at the diagnostic phase soon after the initial visit. We used the following rating scales and neuropsychological tests: Mini-Mental State Examination (MMSE) [39],Clinical Dementia Rating [40], Boston naming test, Vocabulary subtest of the Wechsler Adult Intelligence scale using every second item, Word list learning test [41], Heaton Visual Reproduction test of geometric figures immediately (WMS figures) and after 30 minutes, Story recall, Story recall after 30 minutes, Trail making A test in which a maximum time of 150 s was used, and Verbal fluency PAS test.

#### Alzheimer’s disease patients

Forty-six AD patients fulfilled the diagnostic criteria of AD according to the Diagnostic and Statistical Manual of Mental Disorders (Fourth Edition, Text Revision) (DSM-IV-TR) (American Psychiatric Association (1994) Diagnostic and Statistical Manual of Mental Disorders). Four AD patients had a pathologically confirmed post-mortem AD diagnosis [42]. The AD patients derived from a population-based study database which has been collected at the University of Eastern Finland and described earlier in detail [27], [43], [44].

#### Controls

All controls originated from the population-based study database that included MCI subjects [27], [43]–[45].

#### MCI groups

The subjects with MCI were taken from the Kuopio MCI, a population-based study database gathered at University of Kuopio; they have been described in detail in a previous studies [27], [46]. MCI was diagnosed using the criteria originally proposed by the Mayo Clinic Alzheimer’s Disease Research Center [47], [48]: (1) memory complaint by patient, family, or physician; (2) normal activities of daily living; (3) normal global cognitive function; (4) objective impairment in memory or in one other area of cognitive function as evident by scores >1.5 standard deviation below the age-appropriate mean; (5) clinical dementia rating score of 0.5; and (6) absence of dementia. All the MCI subjects were considered as displaying the amnestic subtype of the syndrome. Those MCI subjects who developed AD during the course of the follow-up were considered as PMCI subjects (n = 16) and those whose status remained stable were considered as having SMCI (n = 48).

Informed written consent was obtained from all subjects according to the Declaration of Helsinki and the study was approved by the ethics committee of The North-Savo Hospital district.

### MRI Acquisition

All MRI images were acquired with one of three different 1.5 T scanners (two Siemens Magnetom Visions, one Siemens Magnetom Avanto, all Siemens Medical Systems, Erlangen, Germany) in the Department of Clinical Radiology, Kuopio University Hospital. In all cases, MRI images were obtained using a T1-weighted 3D MPRAGE sequence. In principle both Siemens Visions provided identical sequences. The resolution parameter differences depended on the project and thus the imaging protocol. The following parameters were used; Magnetom Avanto, with the 3D-MPRAGE: TR = 2400 ms, TE = 3.5 ms, TI = 1000 ms, flip angle = 8°, 160 sagittal slices, matrix 256×256; FOV 300 mm, voxel size = 1.2 mm×1.2 mm×1.2 mm; Magnetom Vision, with the 3D-MPRAGE: coronal slices with TR = 9.7 ms, TE = 4.0 ms, TI = 300 ms, flip angle = 12°, slice thickness = 2.0 mm, field of view = 240×240 mm, matrix size = 256×256, number of slices = 128 and voxel volume = 1.9 mm. Matrix size was always 256×256, but field of view could vary slightly leading to minor variations in the volume of voxel. The large difference in TR between the Vision and Avanto is due to the change in the way that Siemens defined the TR parameter in the MP-RAGE sequence between these two platforms.

All MR images were assessed by an experienced neuroradiologist to exclude subjects with significant alterations such as vascular lesions or severe white matter lesions.

### MRI Analysis Methods

#### Automatic volumetry

Volumes of selected regions of interest were measured using an approach based on fast and robust multi-atlas segmentation [49], [50]. Automatic volumetry analysis was performed in all the regions, but the results were less impressive than with the other methods, and thus we have focussed on the automatic volumetry method in the hippocampus, a well validated biomarker for AD, referred to as Hippocampal volumetry.

In standard atlas-based segmentation, the patient image is segmented in two steps: 1) an intensity template is registered non-rigidly to the patient image and 2) the transformation obtained is used to propagate the tissue or structure labels of the template to patient data. As registrations with real data are not perfect, the use of multiple atlases has been proposed to reduce registration errors. In multi-atlas segmentation, the standard atlas-based approach is applied separately for each atlas and finally the segmentations produced are fused. One common way to fuse segmentations is to select a label that the majority of the atlases predict. In [49], [50], two major extensions to this standard multi-atlas segmentation technique were utilized: atlas-selection and modeling of intensity distributions of patient images. In atlas-selection, only the atlases that are the most similar to the patient image are used, keeping the magnitude of the non-rigid transformations smaller and leading to smaller registration errors. Atlas-based segmentation maximizes some similarity measure between images but does not explicitly utilize the intensity variability of different tissues and structures. To overcome this limitation, the segmentation problem was formulated as a classification problem using the expectation maximization algorithm. The result of the multi-atlas segmentation was incorporated as a spatial prior in the classification.

The atlases used are described in detail in [50]. The atlases were composed of 30 randomly chosen cases from the ADNI cohort: 10 healthy controls, 10 mild-cognitive impairment cases and 10 AD cases. The ADNI web-pages provided hippocampus segmentations which were generated by a semi-automatic procedure described in http://www.loni.ucla.edu/twiki/pub/ADNI/ADNIPostProc/UCSFMRI_Analysis.pdf. The other labels used in the atlases were white matter, grey matter and cerebrospinal fluid (CSF). The tissue segmentations were extracted using a standard automatic segmentation approach [51].

The whole segmentation pipeline is summarized as follows: 1) Register non-rigidly all atlases and a patient image to a template image. One of the atlases was selected as the template image in this work. 2) Select five atlases having the highest normalized mutual information with the patient image in the hippocampus area. The selection is done in the space of the template used in Step 1. 3) Register non-rigidly the patient image to all five atlases. 4) Propagate the all class labels (white matter, grey matter, CSF, hippocampus, background) of the atlases to the co-ordinate system of the patient image and construct a probabilistic atlas for each class, i.e., each voxel contains a probability that it belongs to a certain class. 5) Perform a tissue classification using the expectation maximization algorithm using the probabilistic atlas as a spatial prior. The details of the pipeline have been published [50].

#### Tensor-based morphometry

The TBM analysis was performed using a recently presented multi-template approach [28], [52]. Instead of using only one template to which all the study images would be registered, we used the same 30 randomly selected ADNI images as the template images that were used in the multi-atlas segmentation. Each study image was non-rigidly registered to each template image, and the determinant of the Jacobian matrix (the Jacobian) of the 30 registrations was averaged in logarithmic space for each voxel:
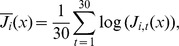
where 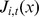 is the Jacobian in voxel *x* for the registration of template *t* to subject *i*. A log transformation was used to make the distribution of the values more Gaussian.

**Figure 1:**
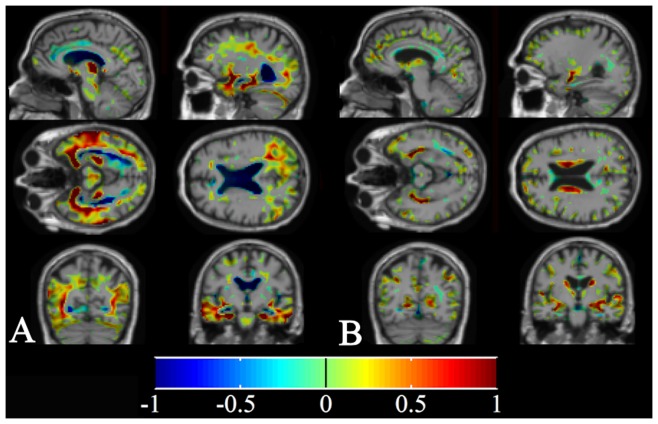
Weight images. Voxels with negative t-values are shown in blue colours and positive values with red colours. The actual weights used are absolute values of the values presented in this visualization. Locations with non-zero weights are shown. *a*. Weights used to compute the TBM features. *b*. Weights used to compute the VBM features.

TBM produces voxel-wise results. In an attempt to end up with a smaller number of features practical for the classification a ROI-wise approach was used. The classification features were computed as a weighted mean of Jacobians within a ROI for each study image from
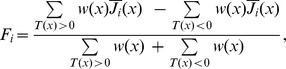
where 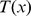 is the t-value of the group-level t-test for voxel *x*, and 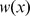 is a weight computed from



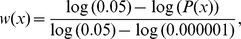
where 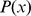 is the p-value of the group-level t-test. In other words, a weighted average was computed separately from voxels in atrophic and expanding areas, their difference was computed, and divided by the sum of the weights. The weights were used to focus the computations on the most important voxels and are visualized in *Figure 1a*. The t-tests were computed using a separate dataset from the ADNI database consisting of randomly selected 100 healthy controls and 100 AD subjects. No covariates were used in the t-test.

The VolumeWarp [49] non-rigid registration tool was used to register images based on local registrations. In this technique, either normalized mutual information, correlation ratio or intensity differences can be used to measure the similarity between images. The curvature of the transformation is used as a regularization term. When intensity differences are being used, then intensity normalization and non-rigid registration steps can be interlaced during the optimization.

#### Voxel-based morphometry

In VBM, the grey matter was segmented from the MRI images using expectation maximization algorithm [51]. A template image, a mean anatomical template of the 30 ADNI images presented above [28], was then non-rigidly registered with the study images, and the grey matter segmentations were propagated to the template space. Finally, the grey matter segmentation was smoothed with a Gaussian filter (FWHM = 4.7 mm), and the mean grey matter concentration was computed for each structure. In addition, modulation [34] was performed for the VBM data, in which the grey matter concentration is multiplied by the Jacobian. In this case, the actual grey matter volume was measured instead of the grey matter concentration. As in TBM, the results of the VBM analysis are obtained at the voxel-level. The ROI-wise mean values were computed as in the TBM analysis, but the Jacobian was replaced by the grey matter concentration, no log transformation was performed, and separate t-tests were performed with VBM data to establish the weights *w(x)* (*Figure 1b*).

### Classification Methods

The Hammers’ atlas of 83 structures was used to define the ROIs in this study. However, our previous studies [28], [29] have shown that if too many ROIs are used the classification accuracy will decrease because the classifier will over-learn the training set. Consequently, we performed an initial study with the same data presented above. This study was performed using all of the 83 ROIs and whole brain measures, and a set of ROIs that gave, on average, the best classification accuracies, along with an evaluation of those areas studied in the literature for AD and FTD [24], [53], [54], i.e. all of these were selected for discriminating between controls, AD, and FTD groups. As the same data were used to select the ROIs and perform the classification study it may be that the selections made were favorable to this specific set of subjects. However, different test and training sets were used in the selection of ROIs and in the classification study which should diminish the bias. The ROIs selected were 1) hippocampus and amygdala, 2) posterior temporal lobe, 3) lateral ventricle in frontal horn, central part and occipital horn, 4) lateral ventricle in temporal horn, 5) gyri hippocampalis et ambiens, 6) anterior cingulate gyrus and 7) superior frontal gyrus.

Three feature values were computed for each ROI; 1) the volume of the ROI, 2) the weighted mean log-Jacobian value for the ROI from the TBM analysis, and 3) the weighted mean grey matter concentration for the ROI from the VBM analysis. The same ROIs were used both in VBM and TBM analyses. In addition, pure left and right hippocampus volumes were computed as they are standard measures for AD.

It is well-known that the volumes of brain structures are dependent on the subject’s age, gender and intracranial volume. Consequently, the feature values were corrected for age, gender, and intracranial volume using a regression model computed from 115 randomly selected healthy subjects from the ADNI database [55], [56]. Only healthy subjects were used in order to guarantee that no disease specific variation would be removed from the data. The corrected values had zero mean and unit standard deviation.

The corrected feature values were then used to classify the data. The classifications were performed between each pair of groups (i.e., controls (C) vs. AD, C vs. FTD, C vs. PMCI, C vs. SMCI, AD vs. FTD etc.). Classifications were done first using individual ROIs so that the best ROIs could be found, and then using simultaneously all the ROIs to obtain an optimal classification accuracy.

A regression classifier was selected as a classifier, and in the case of a multi-ROI classification, a sequential stepwise feature selection was used to avoid overlearning. A dataset was randomly divided into a training set (90% of subjects) and test set (10% of subjects). The feature selection was performed and the classifier was trained with the training set, and the classifier was then applied to the test set and the classification accuracy was computed by comparing the results to the known diagnoses. This was repeated 1000 times, and the classification accuracies of the 1000 unique repetitions were averaged.

There were notable imbalances in the sizes of the study groups. In order to guarantee that the classifier was actually classifying the data based on the classification features and not simply assigning the class of the largest study group to all the subjects, synthetic samples were generated from the smaller group using the Synthetic Minority Over-sampling Technique method [57] so that an equal number of samples could be formed from both study groups. The Synthetic Minority Over-Sampling Technique algorithm generates new samples as a linear combination of two randomly selected close-by samples, where the weighting of the two samples is random.

### Statistical Analysis

The statistically significant differences in demographic data, clinical data and hippocampal volumes between study groups were analyzed by using IBM SPSS 19.0. Differences in gender were assessed with the Chi-Square test and differences in age, MMSE scores and brain tissue volumes using analysis of variance (ANOVA) corrected for multiple comparisons using Bonferroni post-hoc test. Differences in education were analyzed using the non-parametric Kruskal-Wallis test. The level of significance was set to p<0.05.

## Results

### Clinical Data

The demographical and clinical data for the study groups are shown in *Table 1*. The groups did not differ in gender (Pearson Chi-Square 0.257, p>0.05) or years of education (Kruskal-Wallis analysis p>0.05). A one-way ANOVA analysis of age detected significant differences between groups. [F(4,168) 10.3, p<0.0001]; in the post hoc analysis, the FTD group differed from all other groups (p = 0.02 between FTD and PMCI, and p<0.0001 between FTD and the rest of the groups). The FTD patients were significantly younger than subjects in the other groups; the other groups did not differ in terms of age. The MMSE scores differed significantly across the groups, as shown by a one-way ANOVA test. [F(4,157) 24.9, p<0.0001]. The MMSE scores were lower for FTD (p<0.0001) and AD groups (p<0.0001) compared to controls, but no significant difference was found between FTD and AD (p = 0.944).

### Hippocampal Volumes

The hippocampal volumes for the study groups are shown in *Table 1*. Non-normalized hippocampal volumes differed significantly between the groups, as indicated by a one-way ANOVA test [F(4,168) 13.7, p<0.0001].

With non-normalized hippocampal volumes, the FTD (p = 0.004) and AD groups (p<0.0001) had smaller volumes compared to controls. In addition, we detected a significant difference between the control and PMCI groups (p = 0.018). There was a significant difference between FTD and SMCI groups (p = 0.025) and between AD and SMCI (p<0.0001). FTD and AD groups, however did not differ in their hippocampal volumes (p = 0.097), but did differ with one-tailed (p = 0.017) test with Dunnett correction.

Significant results were obtained for normalized hippocampal volumes as well with one-way ANOVA test [F(4,168) 16.7, p<0.0001]. One-way ANOVA corrected for multiple comparisons obtained significant results in the same paired group comparisons as in non-normalized hippocampal volumes.

### Classification Accuracy

**Figure 2:**
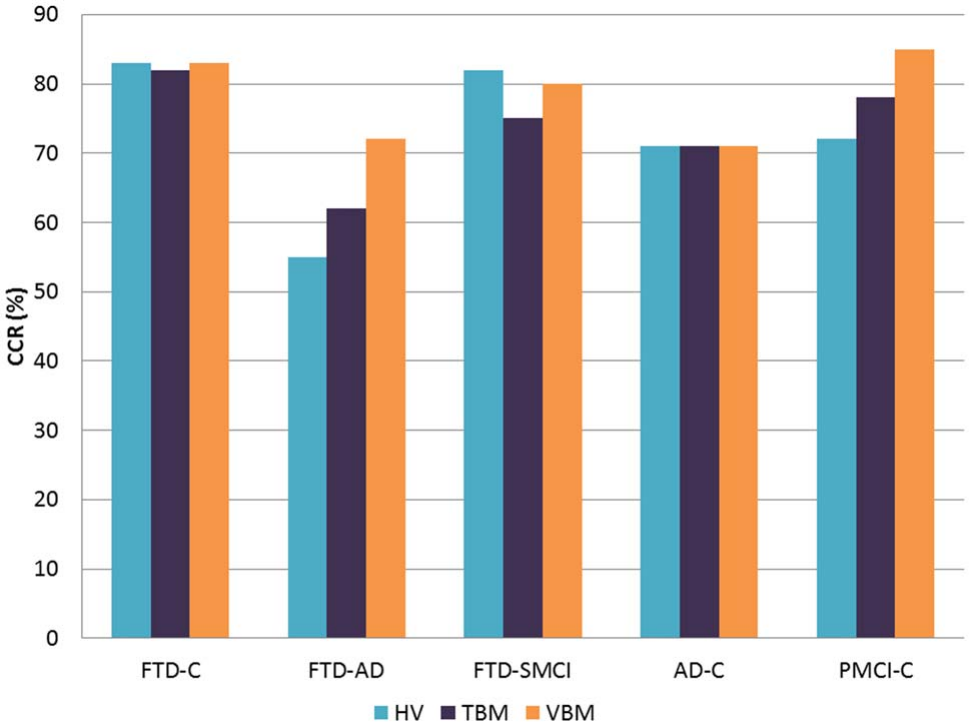
Accuracies (%) obtained using HV, TBM, and VBM for comparisons C vs. FTD, AD vs. FTD, SMCI vs. FTD, C vs. AD, C vs. PMCI.

**Figure 3:**
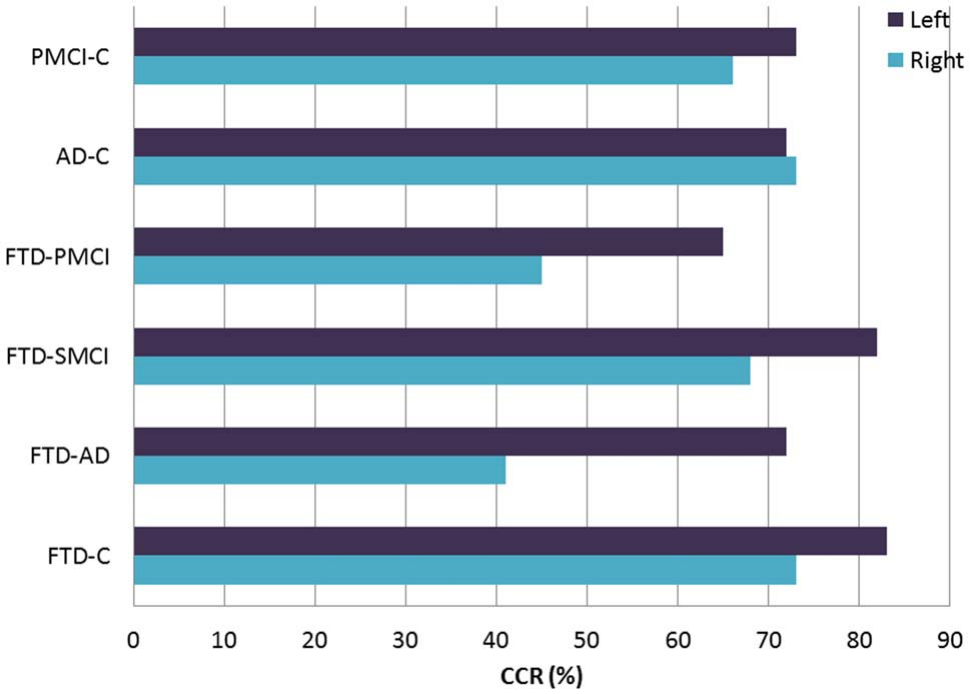
Accuracies (%) of hippocampal volumetry in the following comparisons C vs. PMCI, C vs. AD, PMCI vs. FTD, SMCI vs. FTD, AD vs. FTD and C vs. FTD.

We calculated the Correct classification rate (CCR), CCR standard deviation, Sensitivity (SS) and Specificity (SP) for each individual method first by using all ROIs and then for single ROIs (areas analyzed by each method, one by one). The results of hippocampal volumetry, TBM and VBM using all the ROIs simultaneously are shown in *Table 2* and for single ROIs in *Tables 3* (within the paper), and *4* and *5* (supplementary data). *Figures 2* and *3* illustrate results from *Table 2*.

**Table 2:** Classification using multiple ROIs (all 7 ROIs with feature selection).

	HV			TBM			VBM			VBM modulation		
	CCR	SS	SP	CCR	SS	SP	CCR	SS	SP	CCR	SS	SP
**C vs. FTD**	0.83±0.27	0.84	0.80	0.82±0.20	0.77	0.90	0.83±0.20	0.77	0.91	0.85±0.22	0.82	0.89
**AD vs. FTD**	0.55±0.25	0.55	0.55	0.62±0.24	0.56	0.67	0.72±0.22	0.67	0.76	0.69±0.24	0.66	0.71
**SMCI vs. FTD**	0.82±0.17	0.80	0.84	0.75±0.19	0.69	0.80	0.80±0.17	0.76	0.85	0.76±0.20	0.71	0.80
**PMCI vs. FTD**	0.65±0.36	0.62	0.74	0.58±0.40	0.58	0.61	0.63±0.41	0.63	0.62	0.62±0.40	0.62	0.62
**C vs. AD**	0.71±0.28	0.66	0.79	0.71±0.25	0.63	0.86	0.71±0.27	0.65	0.80	0.74±0.29	0.70	0.80
**SMCI vs. AD**	0.64±0.22	0.62	0.67	0.66±0.21	0.55	0.77	0.65±0.23	0.62	0.66	0.71±0.22	0.71	0.72
**PMCI vs. AD**	0.49±0.39	0.52	0.39	0.47±0.39	0.49	0.41	0.51±0.40	0.54	0.43	0.54±0.40	0.58	0.41
**C vs. PMCI**	0.72±0.28	0.65	0.77	0.78±0.27	0.72	0.82	0.85±0.18	0.72	0.92	0.83±0.23	0.77	0.87
**SMCI vs. PMCI**	0.62±0.23	0.56	0.64	0.74±0.21	0.72	0.75	0.74±0.22	0.60	0.78	0.75±0.20	0.63	0.80
**C vs. SMCI**	0.51±0.34	0.50	0.52	0.60±0.35	0.60	0.60	0.60±0.31	0.56	0.68	0.68±0.32	0.68	0.67

Table 2–5: HV = Hippocampal Volumes; TBM = Tensor-based morphometry; VBM = Voxel-based morphometry; CCR = Correct Classification Accuracy; SS = Sensitivity; SP = Specificity; C = Control; SMCI = Stable Mild Cognitive Impairment; PMCI = Progressive Mild Cognitive Impairment; AD = Alzheimer’s disease; FTD = Frontotemporal Dementia. CCR expressed as mean ± standard deviation.

We also performed Automatic volumetry analysis in all the regions, but the results were less impressive than with the other methods, and thus we have focussed on the automatic volumetry method in the hippocampus, on TBM and on VBM. In addition, we enabled asymmetry features, but no improvement was obtained with these supplements, as compared to the results obtained using the features presented above.

#### Classification using hippocampal volumetry

The total hippocampal volumes achieved a high accuracy in differentiating controls from FTD (CCR = 0.83/SS = 0.84/SP = 0.80) and SMCI from FTD (CCR = 0.82/SS = 0.80/SP = 0.84) (*Figure 3*, *Table 2*). When analyzing right and left side separately, left hippocampal volumes separated FTD from controls with a high accuracy (CCR = 0.83) and also SMCI from FTD (CCR = 0.82) (*Table 3*), while right hippocampal volumes yielded CCR of 0.73 to separate FTD from controls and CCR 0.68 from SMCI vs. FTD. Instead, differentiation between AD and FTD was poor, achieving a low CCR for total hippocampal volume of 0.55.

**Table 3:** Hippocampal volumes.

	Right			Left		
	CCR	SS	SP	CCR	SS	SP
**C vs. FTD**	0.73±0.30	0.72	0.75	0.83±0.27	0.84	0.80
**AD vs. FTD**	0.41±0.25	0.37	0.44	0.56±0.24	0.56	0.56
**SMCI vs. FTD**	0.68±0.21	0.67	0.69	0.82±0.17	0.79	0.84
**PMCI vs. FTD**	0.45±0.40	0.45	0.45	0.65±0.36	0.62	0.73
**C vs. AD**	0.73±0.30	0.71	0.77	0.72±0.27	0.67	0.80
**SMCI vs. AD**	0.67±0.23	0.66	0.68	0.69±0.22	0.64	0.74
**PMCI vs. AD**	0.50±0.40	0.52	0.45	0.54±0.40	0.54	0.54
**C vs. PMCI**	0.65±0.31	0.61	0.68	0.73±0.28	0.66	0.77
**SMCI vs. PMCI**	0.61±0.24	0.61	0.62	0.66±0.22	0.63	0.68
**C vs. SMCI**	0.53±0.35	0.53	0.53	0.54±0.35	0.54	0.53

Left side showed a higher accuracy than right side for all other group comparisons, except for AD vs. C, where left and right yielded similar accuracies.

#### Classification using TBM

Multi-ROI classification (ROIs obtained by applying feature selection on all the ROIs) differentiated controls from FTD with a high accuracy (CCR = 0.82/SS = 0.77/SP = 0.90) (*Table 2*). Using single ROIs, we found good results in differentiating controls from FTD for hippocampus and amygdala (0.80/0.71/0.94), for lateral ventricle (frontal horn, central part and occipital horn) (0.79/0.72/0.89) and for superior frontal gyrus (0.79/0.77/0.83).

However, differentiation between FTD and AD was low for multi-ROI classification (CCR = 0.62) but for single ROIs, superior frontal gyrus yielded a higher accuracy (CCR = 0.71) (*Table 4*). A similar trend was observed for the comparison between PMCI and FTD (CCR for multi-ROI 0.58, range from 0.38 (hippocampus and amygdala) to 0.74 (superior frontal gyrus). Instead, CCR was 0.75 (SS = 0.69, SP = 0.80) in its ability to separate between SMCI and FTD for multi-ROI classification, and the CCR was very similar in all the single ROIs (ranging from 0.71 to 0.72).

If we used multiple ROIs, then this achieved good results in differentiating controls from PMCI (CCR = 0.78/SS = 0.72/SP = 0.82), and also in hippocampus and amygdala using single ROIs (0.84/0.73/0.91), followed by superior frontal gyrus, where the sensitivity was the highest (SS = 0.87).

When comparing SMCI and PMCI, the highest accuracy was obtained in hippocampus and amygdala (CCR = 0.77/SS = 0.73/SP = 0.78).

#### Classification using VBM

If we used a multi-ROI classification, then controls could be differentiated from FTD with high accuracy (CCR = 0.83/SS = 0.77/SP = 0.91) and in addition, controls from PMCI (CCR = 0.85/SS = 0.72/SP = 0.92) (*Table 5*). By using single ROIs, it was possible to achieve impressive results for differentiating controls from FTD in all the regions studied, this being especially the case for lateral ventricle (frontal horn, central part and occipital horn) (CCR = 0.87/SS = 0.81/SP = 0.96), followed by hippocampus and amygdala (CCR = 0.84/SS = 0.78/SP = 0.91) (*Table 5*). Hippocampus and amygdala displayed also a high accuracy in the following comparisons: controls vs. PMCI (CCR = 0.85/SS = 0.73/SP = 0.93) and SMCI vs. PMCI (CCR = 0.81/SS = 0.68/SP = 0.86).

VBM achieved the highest accuracy in differentiating AD and FTD (CCR = 0.72/SS = 0.67/SP = 0.76) (*Table 2*). With respect to single ROIs, the highest level of accuracy for differentiating AD and FTD could be obtained for lateral ventricle (frontal horn, central part and occipital horn) (CCR = 0.73/SS = 0.68/SP = 0.77) (*Table 4*). When comparing SMCI to FTD, CCR was 0.80 and 0.63 for PMCI vs. FTD in the comparison for all ROIs.

The results for VBM with modulation showed no major differences compared to those without modulation *(* *Table 2* *).*

**Table 4:** Tensor-based morphometry.

	Hippocampus and amygdala			Posterior temporal lobe			Lateral Ventricle (frontal horn, central part and occipital horn)			Superior Frontal Gyrus		
	CCR	SS	SP	CCR	SS	SP	CCR	SS	SP	CCR	SS	SP
**C vs. FTD**	0.80±0.17	0.71	0.94	0.72±0.28	0.67	0.80	0.79±0.22	0.72	0.89	0.79±0.27	0.77	0.83
**AD vs. FTD**	0.50±0.24	0.57	0.45	0.60±0.24	0.63	0.57	0.61±0.24	0.59	0.63	0.71±0.23	0.68	0.73
**SMCI vs. FTD**	0.71±0.19	0.61	0.79	0.71±0.20	0.62	0.78	0.72±0.19	0.69	0.74	0.72±0.20	0.69	0.75
**PMCI vs. FTD**	0.38±0.36	0.40	0.33	0.62±0.38	0.61	0.66	0.64±0.36	0.61	0.73	0.74±0.25	0.67	0.89
**C vs. AD**	0.75±0.14	0.63	0.96	0.68±0.30	0.66	0.73	0.69±0.22	0.68	0.70	0.64±0.24	0.61	0.68
**SMCI vs. AD**	0.70±0.19	0.58	0.82	0.60±0.23	0.59	0.60	0.63±0.23	0.61	0.64	0.59±0.24	0.59	0.59
**PMCI vs. AD**	0.47±0.41	0.45	0.50	0.39±0.36	0.43	0.30	0.40±0.38	0.42	0.37	0.57±0.40	0.55	0.62
**C vs. PMCI**	0.84±0.20	0.73	0.91	0.71±0.29	0.70	0.72	0.75±0.31	0.81	0.71	0.77±0.30	0.87	0.72
**SMCI vs. PMCI**	0.77±0.20	0.73	0.78	0.63±0.23	0.70	0.61	0.65±0.22	0.67	0.64	0.63±0.23	0.69	0.61
**C vs. SMCI**	0.57±0.36	0.58	0.56	0.62±0.34	0.61	0.65	0.54±0.35	0.53	0.53	0.63±0.34	0.62	0.64

## Discussion

The diagnostic guidelines for the research criteria of Alzheimer’s Disease [58] suggest that one can use medial temporal lobe atrophy detected on an MRI scan as a diagnostic feature of early AD in addition to episodic memory impairment. While the assessment of medial temporal lobe atrophy can help to differentiate AD patients from healthy controls with high sensitivity and specificity and also identify those MCI patients who will convert to AD, the differentiation between AD and other dementia diseases is not so straightforward. In addition, when using visual evaluation and manual outlining, it seems that the results still depend on the interpreter and thus there is a need for objective methods. In the present study, we used automatic analysis methods of MRI scans in order to achieve the highest accuracy in differentiating memory disorders, focusing on FTD.

In this study, we have obtained a high accuracy in comparing controls with FTD with every image analysis method, and in differentiating AD from FTD using VBM. Hippocampus and amygdala have shown good accuracy in the differentiation between AD and controls, and on the basis of previous studies, it seems that the evaluation of these structures is useful in the diagnosis of FTD as well. In the attempt to differentiate between AD and FTD, we propose that the measurements of superior frontal gyrus and lateral ventricle (frontal horn, central part and occipital horn) can result in high accuracies when studying local field change with TBM and gray matter concentration with VBM.

**Table 5:** Voxel-based morphometry.

	Hippocampus and amygdala			Posterior temporal lobe			Anterior cingulate gyrus			Lateral Ventricle (frontal horn, central part and occipital horn)			Superior Frontal Gyrus		
	CCR	SS	SP	CCR	SS	SP	CCR	SS	SP	CCR	SS	SP	CCR	SS	SP
**C vs. FTD**	0.84±0.18	0.78	0.93	0.77±0.26	0.73	0.81	0.73±0.26	0.67	0.82	0.87±0.12	0.81	0.96	0.72±0.28	0.67	0.79
**AD vs. FTD**	0.57±0.25	0.55	0.60	0.60±0.24	0.58	0.62	0.68±0.22	0.65	0.71	0.73±0.21	0.68	0.77	0.65±0.21	0.58	0.71
**SMCI vs. FTD**	0.79±0.14	0.68	0.89	0.76±0.17	0.67	0.83	0.81±0.12	0.65	0.93	0.83±0.16	0.77	0.87	0.76±0.16	0.65	0.85
**PMCI vs. FTD**	0.49±0.41	0.46	0.59	0.58±0.40	0.56	0.64	0.63±0.38	0.60	0.72	0.70±0.33	0.66	0.80	0.56±0.41	0.54	0.62
**C vs. AD**	0.76±0.19	0.67	0.92	0.72±0.28	0.69	0.78	0.72±0.27	0.67	0.81	0.63±0.32	0.59	0.69	0.70±0.28	0.65	0.77
**SMCI vs. AD**	0.69±0.19	0.54	0.83	0.64±0.22	0.52	0.75	0.66±0.21	0.59	0.73	0.62±0.23	0.53	0.69	0.64±0.22	0.57	0.71
**PMCI vs. AD**	0.51±0.39	0.57	0.36	0.52±0.42	0.53	0.49	0.61±0.39	0.60	0.62	0.42±0.34	0.49	0.27	0.56±0.41	0.58	0.50
**C vs. PMCI**	0.85±0.17	0.73	0.93	0.77±0.26	0.72	0.81	0.79±0.26	0.75	0.81	0.67±0.31	0.63	0.71	0.77±0.28	0.76	0.78
**SMCI vs. PMCI**	0.81±0.17	0.68	0.86	0.75±0.20	0.62	0.79	0.75±0.20	0.67	0.77	0.66±0.23	0.55	0.69	0.77±0.20	0.72	0.79
**C vs. SMCI**	0.63±0.31	0.60	0.69	0.59±0.30	0.55	0.69	0.63±0.33	0.63	0.64	0.57±0.32	0.53	0.65	0.64±0.28	0.57	0.77

We compared AD, controls, stable and progressive MCI and FTD using hippocampal volumetry, TBM and VBM in order to determine the most accurate method. Our main goal was to find the most accurate method and region or regions of interest that could be used to achieve the differentiation of AD from FTD, of controls from FTD, and also of FTD from MCI subjects of whom the majority will develop Alzheimer in the follow-up.

### HV

The present study confirmed that a decrease in hippocampal volumes can be observed not only in Alzheimer but also in FTD. The differences were significant between FTD and controls, and between AD and controls, while no significant difference was found between FTD and AD. CCR was 0.83 to separate FTD from controls whereas only 0.55 for FTD from AD. These results are in line with earlier findings [26]. Other studies also found that hippocampus exhibited more atrophy in AD [59] compared to FTD, and more atrophy in FTD compared to controls but less compared to AD patients [10], [11].

In our study, the left hippocampus had a higher accuracy for all the comparisons, except from C-AD where the accuracy was slightly higher on the right side of the hippocampus. The left hippocampal volumes differentiated controls from FTD with a high accuracy (CCR = 0.83) and also SMCI from FTD (CCR = 0.82). While one study [10] has stated that hippocampal volume possesses a low sensitivity to discriminate FTD from controls (49%), our study achieved a higher sensitivity (80%).

### TBM

We observed clear differences (CCR = 0.82) between FTD patients and controls by using multiple ROIs, and in hippocampus and amygdala, lateral ventricle (frontal horn, central part and occipital horn) and superior frontal gyrus by using single ROIs. Another study comparing behavioral variant FTD patients and controls [53] in a whole brain analysis detected atrophy in the superior frontal gyrus, amygdala and hippocampus as in our study, although they found also atrophy in different regions compared with our study such in bilateral anterior cingulate gyri. One previous study [60] also reported atrophy in FTD patients in hippocampus at an initial stage, as well as in the orbital and superior medial frontal cortices, progressing to temporal cortices and basal ganglia. In another study, differences between sides were obtained, indicating that the degree of temporal lobe atrophy was more extensive on the left side [61].

### VBM

The highest accuracy in differentiating FTD from controls was obtained in lateral ventricle (frontal horn, central part and occipital horn), followed by hippocampus and amygdala and when comparing Alzheimer and FTD in lateral ventricle (frontal horn, central part and occipital horn), and between AD patients and controls in hippocampus and amygdala. These results are interesting considering that lateral ventricle does not include any grey matter and VBM is measuring grey matter density. These findings can be explained by several factors: 1) due to the smoothing used in the VBM procedure the surrounding grey matter structures spread out to the ROI of lateral ventricle, 2) imperfect registrations cause that there are grey matter voxels inside the ROI, and 3) because of the partial volume effect the voxels in the white matter/CSF boundary are classified as grey matter. In order to produce an efficient classifier these effects have to be systematic within the study groups. Consequently, it can be concluded that there are differences between the FTD group and the other groups in the concentration of the grey matter structures next to lateral ventricle (large differences were found especially close to caudate nucleus and thalamus) and/or in the shape of lateral ventricle that can be seen as systematic registration errors. Detailed shape analysis of lateral ventricle might reveal interesting results on the differences between AD and FTD groups.

Other studies have reported results in line with ours, as in left amygdala when comparing FTD and controls [32], and hippocampus and amygdale when comparing AD and controls [62]–[64]. Areas with common atrophy in Alzheimer and FTD patients were the anterior temporal, posterolateral temporal and dorsolateral prefrontal regions of the left hemisphere [65], which alongside with the low accuracy obtained in the hippocampus and amygdala for FTD-AD in our study emphasizes the difficulty of using the medial temporal lobe as a region which undergoes atrophy in both diseases. Instead, some studies have reported other significant regions such as the posterior cingulate cortex as a significant region for differentiating FTD and AD [66]. Comparing FTD with controls the significant regions were the orbitofrontal gyri [66], [67] and left anterior temporal lobe [32], [68], and in the comparison of controls with AD the medial prefrontal gyri [66] and the posterior cingulate gyrus [62], [64].

Previous follow-up studies in patients with mild cognitive impairment, which have aimed at finding a possible conversion to AD, have detected atrophy in hippocampus, parahippocampal cortex [69], anterior cingulate cortex [70], fusiform gyrus [69], [71] and superior temporal gyrus [71]. In our study, the specificity for differentiating controls from PMCI was achieved in hippocampus and amygdala (0.93), posterior temporal lobe (0.81) and anterior cingulate cortex (0.81).

Another study [72] found greater atrophy in the PMCI group as compared to the SMCI group in the hippocampus, posterior cingulate, and parahippocampal gyrus. Our study conducted a PMCI-SMCI comparison and found a high accuracy in the hippocampus, supporting the use of this area for differentiating between both diseases.

Other studies have included FTD patients with genetic mutations, showing widespread and a severe pattern of grey matter loss in the frontal, temporal and parietal lobes in Progranulin positive patients, whereas in Progranulin negative patients, the pattern of loss was restricted to temporal and frontal lobes [73].

In our study, three cases were C9ORF72 gene mutation carriers. A recent study has reported both cortical and cerebellar atrophy in carriers of this mutation [74]. However, visual evaluation did not reveal any major cerebellar atrophy while there was clear cerebral atrophy in the three mutation carriers.

One of the goals of our study was to investigate the advantages of morphometry methods in Alzheimer’s disease and Frontotemporal dementia, by estimating gray matter density (VBM) or the shape of the brain areas of interest (TBM). We generally achieved more accurate results in VBM compared to TBM and HV. VBM measures more directly the tissue loss than TBM by computing the local amount or density of gray matter. Thus the results for the comparison of AD and Frontotemporal dementia cases (atrophy in the frontal lobe areas, which is a commonly known hallmark in FTD) were as expected. Alternatively, the atrophy in the cortex could be estimated by measuring the cortical thickness. On the other hand, TBM is based on a definition of one-to-one mappings between images through registrations which are known to be challenging for cortical foldings. The strength of TBM is that it allows the measurement of the regional size and shape differences of sub-cortical structures for which one-to-one mappings are more clearly defined.

Moreover some methodological causes could lead to misregistration among both methods. While VBM data are only registered to one template, TBM works as a multi-template, addressing a higher possibility of misregistration, On the other hand, a failed misregistration in VBM creates totally useless features, whereas the averaging of over the 30 templates leads to reasonable feature values even if one or two templates had failed in registration. Furthermore, grey matter density values were smoothed after spatial normalization, thus reducing the impact of misregistration, while the Jacobians were not smoothed in TBM.

Automatic Volumetry has been shown to be a useful tool for studying hippocampal volume instead of manual volumetry in Alzheimer cases [50].

A previous study comparing manual volumetry and VBM for frontal and temporal lobes in FTD and AD cases [66] emphasized that volumetrics could be used as an alternative to VBM. Since our study used Automatic Volumetry instead of manual volumetry, this leads us to conclude that automatic volumetry can be used as an alternative to morphometric methods when it focus on hippocampus area, but for multiple areas of interests, morphometric methods, analyzing grey matter density or shape changes, are more advisable. HV is known to be a powerful method in determining the AD or FTD diagnosis, but not for the differentiation of both because hippocampus is an affected area in both diseases. Several reasons can explain why volumetry in other ROIs did not yield improved results compared with the hippocampus. First, only the hippocampus segmentations of our atlases have been manually verified and corrected by an expert. Segmentations for hippocampus were produced (obtained from the ADNI dataset) using a semi-automatic procedure, whereas the segmentations of the 83 structures were derived using the fully automatic atlas propagation method [75]. Second, Hammers’ 83-structure atlases were used in the fully automatic atlas propagation method. This means that the ROIs are not fully optimal for the given application and they do not fully corresponding with the results in [65].

In this study, the classifications were performed between each pair of groups. For a clinical setting, it could be more advisable using a multi-class classification, so we could define the state that a patient has among all the different diseases. This issue will be examined in our next study using PredictAD Fingerprint tool. This compares groups as a whole, but additionally this tool can be used to assign a certain state to an individual patient.

The differences between studies may be explained by different patient populations, demographics, diagnostic criteria, severity of the disease, possible genetic background in FTD, and MRI analysis methods. In the present study, the average age in Frontotemporal dementia patients was comparable to those examined in other studies [9]. Our study groups did not differ in terms of years of education, as in study by Boccardi et al. [9]. We found no significant differences in MMSE scores between AD and FTD patients, whereas in some other studies, FTD patients have scored significantly lower MMSE scores than Alzheimer patients [11].

Our study has some limitations. The sample sizes are relatively small in the different groups. In addition, the FTD group was analyzed as a single group due to the low numbers presenting with the different clinical syndromes. Moreover, only a few cases had autopsy confirmation of the diagnosis. However, the FTD cases included were supervised through clinical long-term follow-up by an experienced neurologist. In one study, the findings have been supported by autopsy in behavioral variant FTD and AD patients [76]. In that study, the authors considered one limitation of their study with autopsy diagnoses was that they lack a validation system that could match mixed pathologies, such as vascular changes, to the atrophy pattern and that their control subjects were older than the dementia cases.

Another limitation in our study is the selection of ROIs, since these 7 regions have been selected based on the same data, which may indicate that the results are not objective. However, different cross-validations were used with different training and test sets being used. Nevertheless, this might have introduced some bias to the results.

In addition, one limitation in our study is the use of different scanners. However, a recent VBM study conducted with multiple scanners and software versions indicated that this represents a minor issue and it also can be overcome by possible pooling of the data [77]. Our previous study using the same scanners [78] detected no differences in the results between the scanners. Therefore we decided not to include the different scanners as a parameter. It is a clinical reality that the scanners are upgraded as are the software versions, although often these confer very minor differences in the actual values of the sequence parameters. However, only robust analysis methods tolerating these minor differences in the pooled data can be advocated when selecting the tools for use in the clinic in the differentiation between dementia diseases.

### Conclusion

From all the methods investigated, the results which achieved the highest accuracy for differentiating FTD and AD were clearly obtained by using gray matter density analysis with VBM, which can be considered as a useful tool for differentiating between these dementia diseases. HV and TBM remain as useful methods for differentiating FTD patients from C and the MCI stages, with as good results as those obtained with VBM. Lateral ventricle (frontal horn, central part and occipital horn) are regions of interest which show the highest accuracy, followed by anterior cingulate gyrus and superior frontal gyrus, all of them with high specificities.
